# Augmented Reality Technology as a Teaching Strategy for Learning Pediatric Asthma Management: Mixed Methods Study

**DOI:** 10.2196/23963

**Published:** 2020-12-02

**Authors:** Suhasini Kotcherlakota, Peggy Pelish, Katherine Hoffman, Kevin Kupzyk, Patrick Rejda

**Affiliations:** 1 College of Nursing University of Nebraska Medical Center Omaha, NE United States

**Keywords:** augmented reality, graduate nursing, pediatric asthma management, flipped learning, nursing, asthma, chronic disease, nurse practitioner, nursing students, pediatric asthma

## Abstract

**Background:**

Asthma is a major chronic disease affecting 8.6% of children in the United States.

**Objective:**

The purpose of this research was to assess the use of clinical simulation scenarios using augmented reality technology to evaluate learning outcomes for nurse practitioner students studying pediatric asthma management.

**Methods:**

A mixed-methods pilot study was conducted with 2 cohorts of graduate pediatric nurse practitioner students (N=21), with each cohort participating for 2 semesters.

**Results:**

Significant improvements in pediatric asthma test scores (*P*<.001) of student learning were found in both cohorts at posttest in both semesters. Student satisfaction with the augmented reality technology was found to be high. The focus group discussions revealed that the simulation was realistic and helpful for a flipped classroom approach.

**Conclusions:**

The study results suggest augmented reality simulation to be valuable in teaching pediatric asthma management content in graduate nursing education.

## Introduction

### Background

Asthma is a major chronic disease affecting 8.6% of children in the United States [1]. Nurse practitioners play a vital role in assessing and effectively managing asthma. Educating nursing practitioner students and empowering them with relevant clinical health information can improve pediatric asthma care delivery. Furthermore, the US Department of Education [2] advocates that educators enable learning through technology.

This study examined the use of augmented reality (AR) technology in teaching clinical simulation content. AR technology is emerging, and the earliest evidence of its use in the classroom and simulation settings or during independent and asynchronous activities suggests that this pedagogical approach is effective in transforming nursing education [3]. AR technology consists of augmenting layers of sounds, images, and videos into real-life environments.

Results from a study by Aebersold et al [4] in nursing and medical education showed enhanced health care learning and critical thinking when providing a more authentic and engaging experience. Integrating simulation through AR for health care education has been implemented with positive results. Data from 69 students who completed a merged learning experience showed that 86% preferred the augmented virtual simulation experience over the traditional simulation experience [4]. We searched the Google Scholar, JMIR, and literature databases with the search terms “augmented reality” AND “clinical simulation” AND “nursing” AND “graduate” for papers written since 2016 and found few comparable studies, with only 1 study in graduate education. A study reported that several studies in science, engineering, and health fields used AR with school students, undergraduate students, or others. Still, only 1% of studies used graduate students as their sample group [5]. Studies have shown that handheld devices, such as iPads, tablets, or glasses, are preferable for delivering AR education because of the affordability and portability they offer [6-8]. AR education offered via mobile devices is adequate for conveying auditory and visual information compared with the increased cognitive demands associated with immersive technologies (and experiences) [4,9]. A critical implication of this modality type is the feasibility for distance learning and telehealth situations [10]. Using iPads, quick response (QR) codes, and the Augmented Reality and Interactive Storytelling (ARIS) mobile app [11], the Augmented Reality Integrated Simulation Education (ARISE) project team developed 150 clinical scenarios [12] for health care students [13]. These scenarios merge the concepts of simulation with AR. The developers replicated realistic storylines in the scenarios with features such as videos and pictures of clinic settings, as well as patient introductions, nurse-patient encounters, and live patient simulations. Creating a sense of realism is essential for educators using simulation lab scenarios. These features maximize student learning and provide opportunities for practice learning in a safe environment compared with a live patient interaction, in which risks for mistakes are higher.

The purpose of this study was to evaluate the use of clinical simulation scenarios using AR technology with nurse practitioner students studying pediatric asthma management.

### Research Questions

Our research questions were (1) Does the AR simulation improve student learning outcomes related to pediatric asthma management, and are there differences in the 4 factors (attention, relevance, confidence, and satisfaction) that measure student motivation when using AR technology to learn pediatric asthma management content? and (2) What are the perceived benefits and difficulties of using the AR technology to learn the pediatric asthma management content?

## Methods

### Study Design

A mixed-methods single-condition study was conducted with 2 cohorts of graduate nursing students [14]. Each cohort participated for 2 semesters (fall and spring). ARISE clinical scenarios (Multimedia Appendices 1 and 2) were used in a flipped classroom approach, in which students interacted with the clinical scenario independently and discussion occurred in class. Students used the AR ARIS app on their iPad to prioritize what to do first, as though in a patient care setting. This process allowed for making connections between classroom learning and clinical practice. The institutional review board decided the study was exempt because it was a program evaluation.

### Sample

The sample consisted of 21 students enrolled in 2 courses in the pediatric nurse practitioner program at a large Midwestern US university. There were 12 students in the first cohort (2018-2019) and 9 in the second cohort (2019-2020); 19 were women and 2 were men. Hertzog [15] recommends 20 to 25 participants to demonstrate efficacy in single-group studies.

### Measurements

A pretest and posttest of knowledge about pediatric asthma was used to assess student learning. At posttest, data were collected using 3 reliable and valid measurements. The Instructional Materials Motivation Survey (IMMS) has 4 subscales that measure motivational factors: attention, relevance, confidence, and satisfaction (ARCS) [16]. Items on the IMMS are on a 5-point scale ranging from 1 (not true) to 5 (very true). The 20-item Simulation Design Scale (SDS) has 5 subscales that rate the importance of objectives and information, support, problem solving, feedback and guided reflection, and fidelity (realism) design elements [17]. Item responses on the SDS range from 1 (not important or strongly disagree) to 5 (very important or strongly agree). The 13-item Student Satisfaction and Self-Confidence in Learning Scale (SSSC) has point scales ranging from 1 (strongly disagree) to 5 (strongly agree) and measured attitudes toward simulation [18]. All rating scale scores have well-established psychometric properties, with all subscale and total scores having Cronbach α reliability coefficients above .80.

### Procedure

All students received an iPad (Wi-Fi connectivity compatible) and the accompanying ARIS app from the university for the duration of the study. Each semester, students took the pretest. A 4-week window was given to complete the pediatric asthma simulation on the iPad, which was followed by the 3 measurements and the posttest. A structured focus group interview was then conducted to elicit student perceptions of the benefits and difficulties associated with the learning experience.

### Data Analysis

Descriptive statistics were calculated on all variables and 2-tailed dependent samples *t* tests were performed to assess student learning of pediatric asthma by analyzing the change from pretest to posttest each semester. Correlations were used to assess motivational factors associated with student learning. IBM SPSS version 25 (IBM Corp) was used for all analyses, and a .05 α level was used to determine statistical significance.

## Results

### Student Learning Outcomes

**Figure 1:**
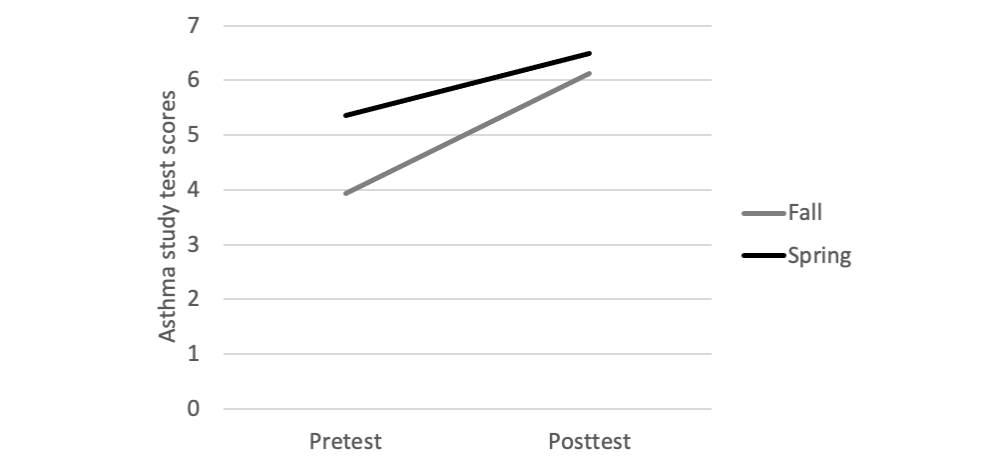
Comparison of asthma study pretest and posttest scores in the fall and spring semesters.

Significant improvement in pediatric asthma test scores of student learning were found in both cohorts at posttest in both the fall semester (t_20_=8.95; *P*<.001) and spring semester (t_16_=4.35; *P*<.001), as shown in Figure 1. The test scores indicated that the AR allowed for learning related to the assessment and management of asthma. No studies for comparison were located in graduate nursing didactic education.

### Factors Associated With Student Learning

Overall data from the IMMS and SDS measurements and the subscale scores were reviewed (see Table 1).

Average scores ranged from 3.2 to 4.2, all above the midpoint (3.0) of the subscales, indicating that students were generally motivated, satisfied, and confident with their learning and felt the content was important. Student learning outcomes, represented by increases in pediatric asthma test scores during a semester, were not significantly correlated with any of the factors. There were small to moderate correlations observed, and significant correlations were observed among several factors. There were no significant differences on the ARCS motivation factors in the IMMS scale in either cohort of students between the fall and spring semesters, but pretest motivation was high and did not decline. Average scores were 3.8 for helpfulness and enjoyability of the teaching methods. Student satisfaction with current learning (SSSC) was found to be highly positively correlated with students’ motivation and confidence (IMMS) in both semesters (*r*=0.50 to *r*=0.67; *P*<.001 to *P*=.046).

**Table 1:** Descriptive statistics (means and standard deviations) of scored measures.

Measurements		Fall, mean (SD)	Spring, mean (SD)
**Simulation Design Scale^a^**			
	Objectives and information	3.73 (0.55)	3.81 (0.83)
	Support	3.94 (0.73)	4.06 (0.66)
	Problem solving	3.82 (0.65)	4.08 (0.47)
	Feedback and guided reflection	3.77 (0.43)	3.89 (0.51)
	Fidelity (realism)	4.38 (0.52)	4.35 (0.46)
**Student Satisfaction and Self-Confidence in Learning^b^**			
	Satisfaction with current learning	3.78 (0.59)	3.84 (0.46)
	Self-confidence in learning	3.86 (0.46)	3.82 (0.42)
**Instructional Materials Motivation Survey^c^**			
	Attention	3.72 (0.68)	3.7 (0.42)
	Relevance	3.71 (0.68)	3.69 (0.51)
	Confidence	3.9 (0.54)	4.11 (0.52)
	Satisfaction	3.19 (1.03)	3.06 (0.72)

^a^SDS scale ranges from 1 (not important or strongly disagree) to 5 (very important or strongly agree).

^b^SSSC scale ranges from 1 (strongly disagree) to 5 (strongly agree).

^c^IMMS scale ranges from 1 (not true) to 5 (very true).

## Discussion

### Summary

The positive correlations among students’ motivation, satisfaction, and confidence are a clear indication for faculty that using AR technology in their teaching approach may lead to successful learning outcomes and experiences. These study results are consistent with the finding that students perceived high ease of use and motivation for using AR pedagogy on mobile devices [19]. Future use of QR codes to experience simulated sound recordings could be used in a simulation environment.

### Benefits and Difficulties

Focus group leaders asked students questions about the benefits of the AR, such as how the simulation matched the intended pediatric asthma management content, how the simulation fostered confidence in assessment, and what learning pearls they gained from the activity. Leaders asked about difficulties in using the iPad and links to the simulation and what was hard to access. An additional question asked about the future use of iPad learning. Students found the simulation realistic and said that it tied together teaching and learning. However, they struggled with iPad passwords and scanning the simulation codes. They expressed concerns related to using a device that they did not own. Those who already had a personal iPad would have preferred to use their own device. In each cohort, students had fewer questions in the spring than in the fall. Students recommended providing iPads with simulation software already installed. They discussed their difficulty accessing the ARIS app and the appropriate simulation. A suggestion was made to create a series of screenshots to use as guidelines. Once they overcame that problem, students found the simulation realistic and consistent with pediatric asthma management practices. They believed the app could be expanded to include other lung sounds and medical conditions.

### Limitations

The study was conducted in a single school location with no control group and a small sample size. Focus groups were conducted by course faculty. Comparable groups using the previous mode of instruction in the courses would have added to the external validity of the results.

### Conclusion

Overall, AR technology is promising for simulation in graduate nursing education. AR simulation provided a flexibly timed pediatric situation that improved student learning about pediatric asthma management and aided transfer of knowledge to clinical practice. This study demonstrated the use of technology to provide clinical situations without the concerns related to clinical site placements. Simulation plus AR allowed for realistic pediatric patient assessment. Student comments implied the need for support and guidance using technology devices.

This study advocates that AR technology can be effectively administered in nursing instruction, making it possible for faculty to spend less class time presenting facts and procedures and more time engaging students in quality debriefing. The findings add to our understanding of expanding AR use to other nursing concepts in flipped or emergency remote teaching modalities (see Multimedia Appendix 3).

## Appendix

Multimedia Appendix 1 Asthma Simulation Nursing L1 UNMC version.

Multimedia Appendix 2 Asthma Simulation Nursing L2 UNMC version.

Multimedia Appendix 3 Example technology implementation considerations prior to using augmented reality technology for instructional activity.

## Abbreviations

AR: Augmented Reality
ARCS: Attention, Relevance, Confidence, and Satisfaction
ARIS: Augmented Reality and Interactive Storytelling
ARISE: Augmented Reality Integrated Simulation Education
IMMS: Instructional Materials Motivation Survey
SDS: Simulation Design Scale
SSSC: Student Satisfaction and Self-Confidence in Learning Scale
QR: Quick Response
