# Supplementary material for: Augmented Reality Technology as a Teaching Strategy for Learning Pediatric Asthma Management: Mixed Methods Study
Source: JMIR Nurs. 2020 Dec 2;3(1):e23963. doi: 10.2196/23963 (PMC8373372; doi:10.2196/23963)
Supplement: Multimedia Appendix 1 [file nursing_v3i1e23963_app1.docx]

**PEDIATRIC ASTHMA Level 1**

**(UNMC CON Edited version)**

**For this study using the iPAD, you will need to configure the device using the UNMC instruction sheet.**

**The next step is to download the app related to the ARIS stimulations. This can be downloaded from the App Store:** [**http://itunes.apple.com/us/app/aris/id371788434**](http://itunes.apple.com/us/app/aris/id371788434)

**When it asks for your credit card, you can respond “No credit card.”**

**For help with the iPAD, contact Patrick Rejda at** [**prejda@unmc.edu**](mailto:prejda@unmc.edu)

**You can access the NRSG 671 course from your iPAD to do the Asthma study Pre-test Quiz which has 7 questions. Then follow the case in this simulation. After that is completed, you will need to take the Asthma study Post-test Quiz which will be available until Sept 26, 2017.**

Estimated Time: 30 minutes • Debriefing Time: 60 minutes on October 3, 2017 in class

| LEARNING OBJECTIVES |
| --- |

1. Obtain a health history
2. Perform a general survey assessment
3. Perform a basic respiratory assessment
4. Recognize and report deviation from norms
5. Accurately document findings

| SIMULATION LEARNING ENVIRONMENT |
| --- |

| **PATIENT PROFILE** |  |
| --- | --- |
| Name: Patrick A. Armstrong | Height: 177.5 cm (5 ft 11 in) |
| DOB: 11/16/20xx | Weight: 109 kg (240 lbs) |
| Age: 16 | Code Status: Full code |
| MR#: 1116 | Primary Language spoken: English |
| Gender: Male | Allergies: NKDA |

**Patient**

- Street clothes, ball cap, has his phone
- Has his albuterol inhaler with him

**Vital signs taken when brought into the clinic**

- Vitals: HR 64, RR 14, BP 108/64, Temp 36.8, O2 sat 100% on RA, Pain 0/10

**Student considerations at the beginning of the simulation:**

- “Meet Your Patient” (on iPad) will explain how the iPad works in the simulated learning environment including:
- There are multiple QR codes in the simulation, but you should only scan them if you think it will provide data necessary for assessment and evaluation of the patient.
- For the most authentic lung sound experience, you can use your stethoscope for all QR Lung Codes.
- Medications are underlined and hyperlinked to DailyMed, which is a medication reference housed by the National Library of Medicine. You can click on these links during the simulation for up-to-date medication content, labels, and package insert information.
- You can view a sample Protocol that is typically found in this setting

| 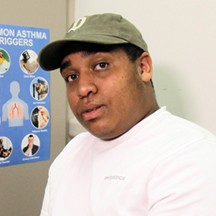 | Scan to Begin  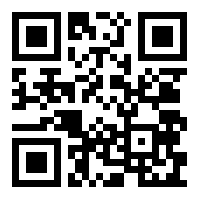 |
| --- | --- |

Patient Name: Patrick A. Armstrong

SCENARIO OVERVIEW

Patrick Armstrong is a 16-year-old patient who presents to a clinic for a routine follow-up visit for asthma. EMR forms are consistent with information usually provided in a clinic setting. He is in stable condition, but in the “yellow zone” on the Asthma Action Plan. Students should perform a focused respiratory assessment using QR codes to simulate various anatomical locations, gather focused subjective data, and document their findings.

Now that you have met your patient, consider these steps in the assessment of Patrick:

1. What are your clinical concerns when you hear that a patient has asthma?
2. You would begin with the subjective interview:

Patient is distracted by the messages coming in on his phone. He doesn’t really want to be at the appointment and is not motivated to learn more about his asthma. He tells you “I’m not sure why I have to be here again.”

You ask:

Do you feel short of breath today? Answer: “A little.”

Do you have a cough? Answer: “At night sometimes.”

Do you take any other medications? Answer: “Just Advil when I’m sore from football practice.”

Do you smoke? Answer: “Sometimes”

Does anyone in your household smoke? Answer: “Yes”

Are you following an asthma action plan? Answer: “Not sure?”

Do you have your inhaler with you? Answer: “Yes”

1. Based the patient’s subjective history, consider what focused assessments do you plan to perform?

**Review the chart items on the left side of the screen for your initial information. Please ignore the input screen for the vital signs. Those do not need to be entered into the chart.**

**QR CODES**

**The QR code on the next page links you to a peak flow meter.**

**The pages after that allow you to link to the lung sounds at each area of Patrick’s case. Please listen carefully so that you can document what you are hearing at each area.**

| QR CODES |
| --- |


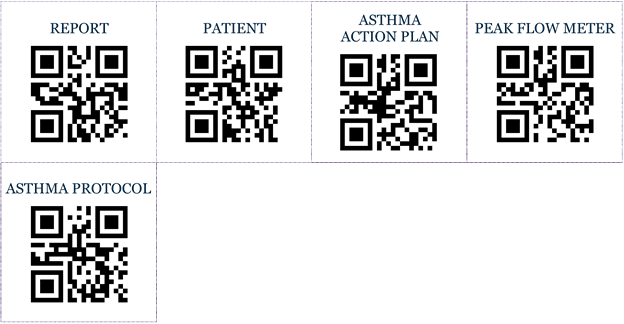


| CHEST QR CODES - ANTERIOR |
| --- |

| 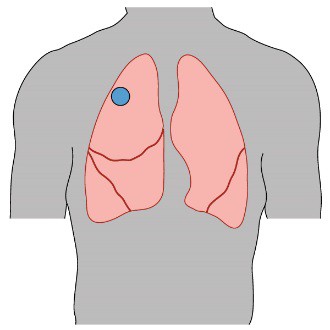 |  | 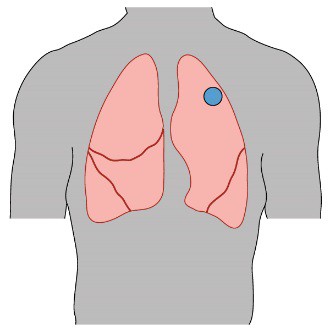 |
| --- | --- | --- |
| ANTERIOR 2  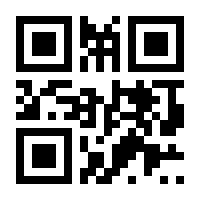 |  | ANTERIOR 3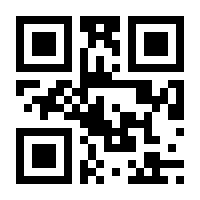 |

| 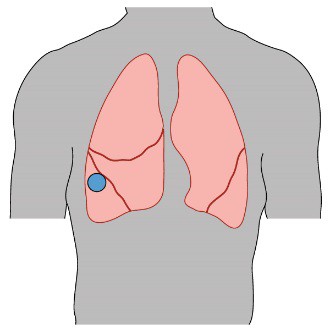 |  | 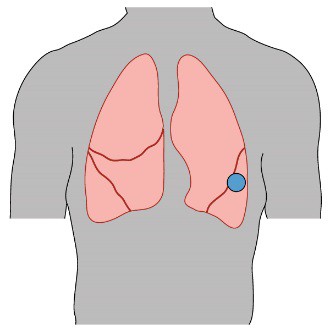 |
| --- | --- | --- |
| ANTERIOR 6  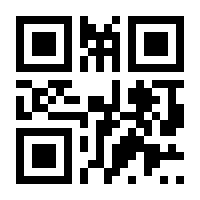 |  | ANTERIOR 7  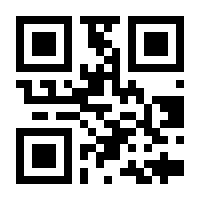 |

| CHEST QR CODES - POSTERIOR |
| --- |

| 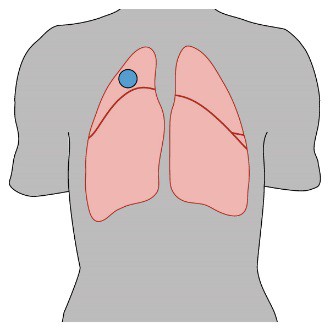 |  | 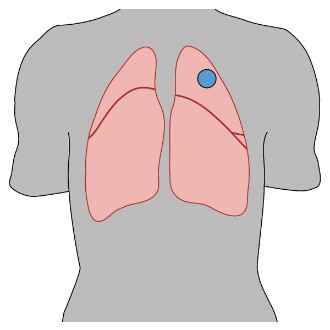 |
| --- | --- | --- |
| POSTERIOR 0  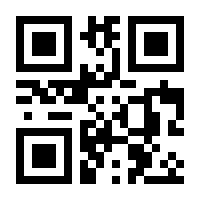 |  | POSTERIOR 1  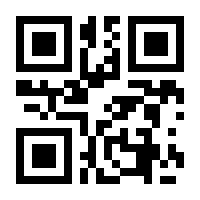 |

| 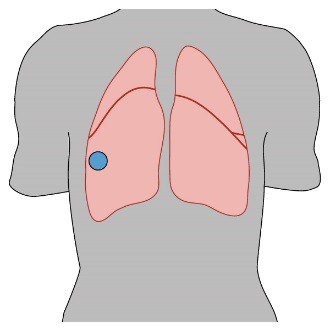 |  | 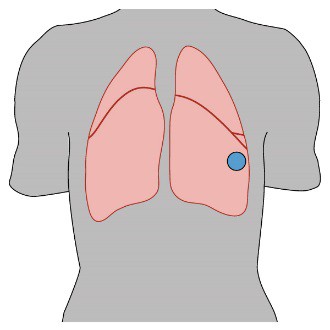 |
| --- | --- | --- |
| POSTERIOR 4  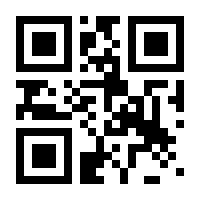 |  | POSTERIOR 5  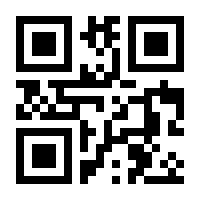 |

| CHEST QR CODES - AXILLARY |
| --- |

| 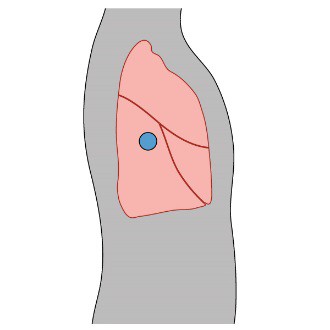 |  | 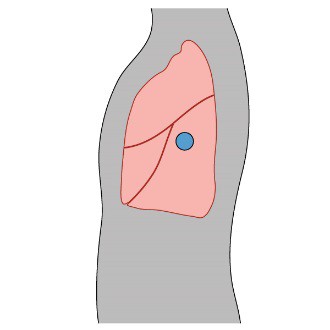 |
| --- | --- | --- |
| RIGHT AXILLARY 1  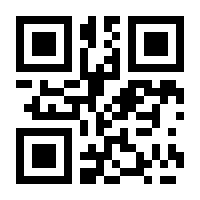 |  | LEFT AXILLARY 1  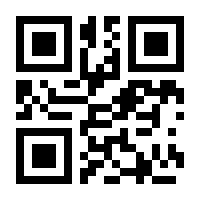 |

| PLAN |
| --- |

Because you have heard adventitious lung sounds, you would recommend his Albuterol medication for treatment in the clinic.

PROBLEM LIST

Asthma exacerbation ICD-10 code: J45.901

MEDICATION LIST

Albuterol inhaler 2 puffs q4 hours PRN for shortness of breath

**Student Considerations at the end of the simulation:**

- How will you modify your approach to the developmental level of teenager?
- How would you describe the lungs sounds you are hearing?
- Analyze the lungs sounds and how they relate to what is occurring in the patient’s lungs?
- View the Asthma Severity Protocol that is often used in a clinic setting. How would you rate Patrick’s current respiratory status using this protocol?
- How is the Asthma Action Plan used to help the patients self-manage their asthma?

**This part of the Asthma iPAD study is completed. There will be a debriefing discuss in class on October 3, 2017.**

**Please return to the NRSG 671 Canvas site to take the Asthma Study Post-test Quiz.**

| CREDITS |
| --- |

Asthma action plan from National Heart, Lung and Blood Institute at <https://www.nhlbi.nih.gov/health/resources/lung/asthma-action-plan>

Asthma severity protocol from: National Heart, Lung, Blood Institute (2007) The Expert Panel Report 3 (EPR–3) Guidelines for the Diagnosis and Management of Asthma.

Downloaded from: [http://www.nhlbi.nih.gov/health-pro/guidelines/current/asthma-](http://www.nhlbi.nih.gov/health-pro/guidelines/current/asthma-guidelines) [guidelines](http://www.nhlbi.nih.gov/health-pro/guidelines/current/asthma-guidelines))

Medication information from National Library of Medicine: Daily Med at <http://dailymed.nlm.nih.gov/dailymed/>

Normal lung sound from Thinklabs Medical, LLC, Centennial, CO at <http://www.thinklabs.com/lung-sounds>

Patient education files adapted from OSCE Skills and wikiHow at [http://www.osceskills.com/e-](http://www.osceskills.com/e-learning/subjects/explaining-the-peak-expiratory-flow-rate-technique/) [learning/subjects/explaining-the-peak-expiratory-flow-rate-technique/](http://www.osceskills.com/e-learning/subjects/explaining-the-peak-expiratory-flow-rate-technique/) and <http://www.wikihow.com/Use-a-Peak-Flow-Meter>

Pictures from Shutterstock.com

Wheeze lung sound from Wikipedia at <https://en.wikipedia.org/wiki/Wheeze>
