# Supplementary material for: Augmented Reality Technology as a Teaching Strategy for Learning Pediatric Asthma Management: Mixed Methods Study
Source: JMIR Nurs. 2020 Dec 2;3(1):e23963. doi: 10.2196/23963 (PMC8373372; doi:10.2196/23963)
Supplement: Multimedia Appendix 2 [file nursing_v3i1e23963_app2.docx]

Multimedia Appendix 2: Asthma Simulation Nursing L2 UNMC version

# PEDIATRIC ASTHMA Level 2

**(UNMC CON Edited version)**

**You can access the NRSG 672 course from your iPAD to do the Asthma study Pre-test Quiz which has 7 questions. Then follow the case in this simulation. After that is completed, you will need to take the Asthma study Post-test Quiz which will be available until Feb 13, 2018.**

## LEARNING OBJECTIVES

1. Perform a focused respiratory assessment on a pediatric patient with asthma
2. Obtain a focused health history
3. Evaluate the patient’s use of a peak flow meter
4. Evaluate the patient’s self-administration of an inhaler
5. Perform patient education using the Asthma Action Plan

NURSING | LEVEL: 2

1. Determine new plan of care for the patient.

| SIMULATION LEARNING ENVIRONMENT |
| --- |

Scan to Begin

NURSING | LEVEL: 2


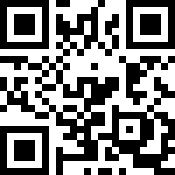


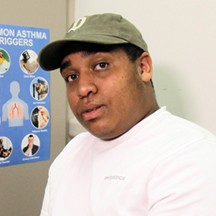
Patient Name: Patrick A. Armstrong

## SCENARIO OVERVIEW

Patrick Armstrong is a 16-year-old patient who presents to a clinic for a visit for asthma. His asthma worsened after being exposed to a cat at a friend’s house, so he called for a same day appointment. He is in stable condition but in the “yellow zone” on the Asthma Action Plan.

#### Vital signs: HR 84, RR 24, BP 108/64, Temp 36.8, O2 sat 97% on RA, Pain 0/10

Now that you have met your patient, consider these steps in the assessment of Patrick:

1. What are your clinical concerns when you hear that this patient with asthma has had a worsening of his symptoms?
2. You would begin with the subjective interview:

Overview: Patient is feeling short of breath and understands that the exposure to the cat at his friend’s house triggered his asthma. He has already used his albuterol 4-5 times today.

- Initial patient responses include:
  1. “I was hanging out at a friend’s house today…. playing video games. I started to have… a hard time breathing.”
  2. “I noticed they … have a cat…. I’m allergic to cats.”
  3. “I’ve used my inhaler ……like 4 or 5 times today.”
  4. “I called my dad at work ……and he said to go see the doctor…. so that’s why I’m here.”
- Other questions:

Do you feel short of breath today? Answer: “Yes.”

Do you have a cough? Answer: “At night sometimes.”

Do you take any other medications? Answer: “Just Advil when I’m sore from football practice.”

NURSING | LEVEL: 2

Do you smoke? Answer: “Sometimes. But don’t tell my parents, they’ll get mad.”

Does anyone in your household smoke? Answer: “Yes”

Are you following an asthma action plan? Answer: “Not sure?”

Do you have your inhaler with you? Answer: “Yes”

1. Based the patient’s subjective history, consider what focused assessments do you plan to perform?

**QR CODES**

**The QR code on the next pages link you to a peak flow meter and the lung sounds at each area of Patrick’s case. Please listen carefully so that you can document what you are hearing at each area**

**And how Patrick uses his Peak Flow B at first and the Peak Flow A after his education.**


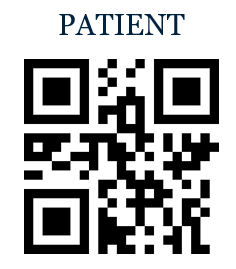


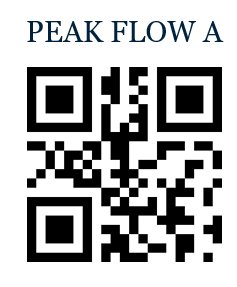

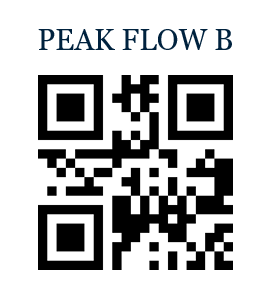


**CHEST QR Codes**

| 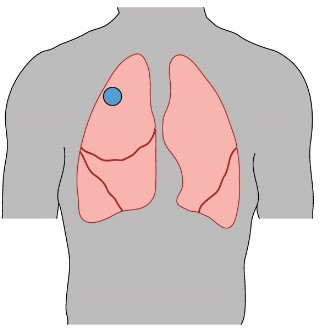 |  | 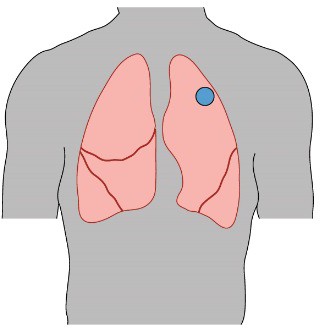 |
| --- | --- | --- |
| ANTERIOR 2  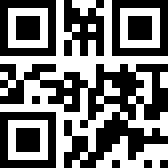 |  | ANTERIOR 3  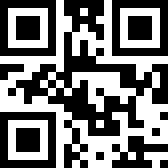 |

| 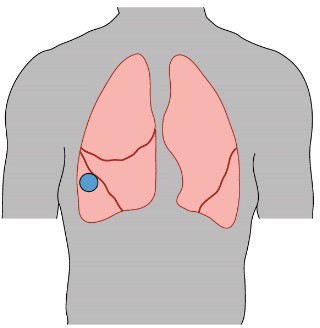 |  | 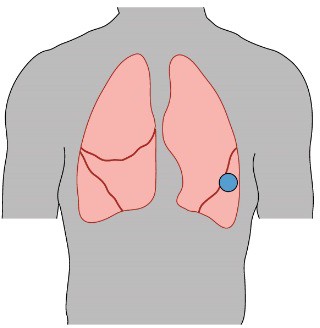 |
| --- | --- | --- |
| ANTERIOR 6  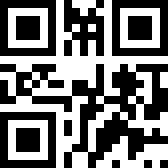 |  | ANTERIOR 7  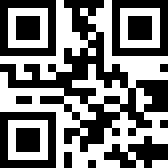 |

**CHEST QR Codes**

| 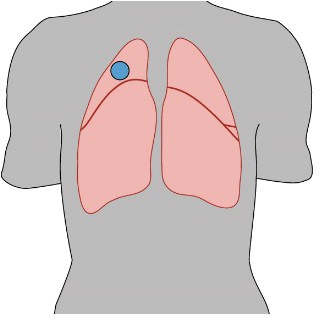 |  | 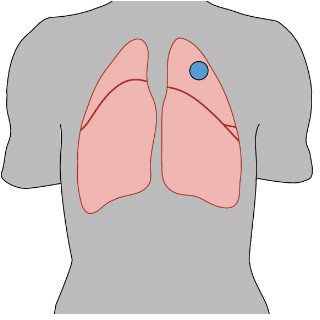 |
| --- | --- | --- |
| POSTERIOR 0  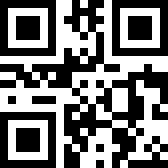 |  | POSTERIOR 1  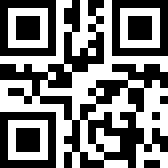 |

| 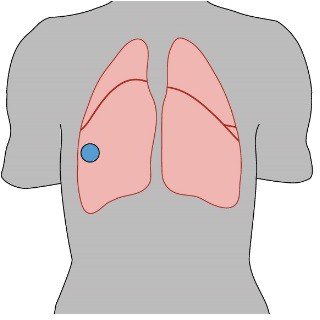 |  | 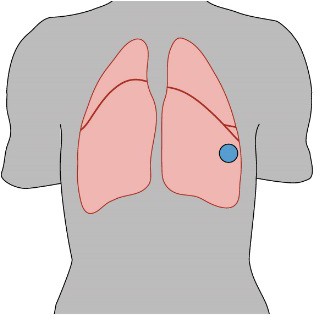 |
| --- | --- | --- |
| POSTERIOR 4  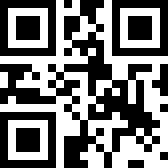 |  | POSTERIOR 5  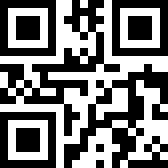 |

| PLAN |
| --- |

Because you have heard adventitious lung sounds throughout, you would recommend a step up of his asthma treatment. Look at this national website for the steps of assessment and care <https://www.nhlbi.nih.gov/sites/default/files/media/docs/asthma_qrg_0_0.pdf>.

Determine the new medication orders and develop a new Asthma Action Plan. Assess his ability to use his inhaler with the examples in Inhaler A and Inhaler C (one shows poor use).

PROBLEM LIST

Asthma exacerbation ICD-10 code: J45.901

TREATMENT ORDER LIST


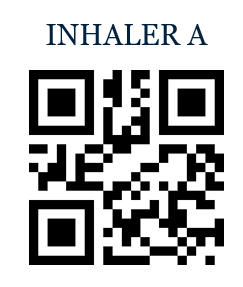

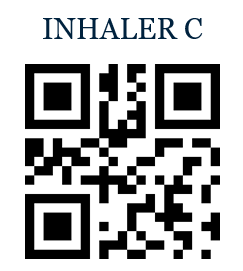


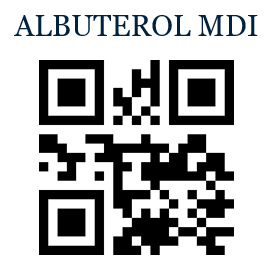

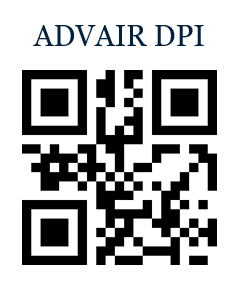


Nebulized albuterol treatment or MDI in the clinic

Reassess after treatment

MEDICATION LIST additions

Advair DPI 2 puffs BI

**Student Considerations at the end of the simulation:**

- How will you discuss the new medication in relation to the current use of Albuterol that is described as a rescue medication only?
- How would you describe the lungs sounds you are hearing?
- Analyze the lungs sounds and how they relate to what is occurring in the patient’s lungs?
- View the Asthma Severity Protocol that is often used in a clinic setting. How would you rate Patrick’s current respiratory status using this protocol?
- How is the Asthma Action Plan changed now?
- How would you teach use of the Peak Flow Meter and the Albuterol MDI? See the link to the Patient Education for suggestions.
- When should he return to the clinic? If the wheezing continues, what new mediation should be considered after you reference the national stepwise treatment plans?

**This part of the Asthma iPAD study is completed. There will be a debriefing discuss in class on March 6, 2018.**

**Please return to the NRSG 672 Canvas site to take the Asthma Study Post-test Quiz.**

NURSING | LEVEL: 2

**CREDITS**

Asthma action plan from National Heart, Lung and Blood Institute at <https://www.nhlbi.nih.gov/health/resources/lung/asthma-action-plan>

Asthma Severity protocol from: National Heart, Lung, Blood Institute (2007) The Expert Panel Report 3 (EPR–3) Guidelines for the Diagnosis and Management of Asthma.

NURSING | LEVEL: 2

Downloaded from: [http://www.nhlbi.nih.gov/health-pro/guidelines/current/asthma-](http://www.nhlbi.nih.gov/health-pro/guidelines/current/asthma-guidelines) [guidelines](http://www.nhlbi.nih.gov/health-pro/guidelines/current/asthma-guidelines))

Medication information from National Library of Medicine: Daily Med at <http://dailymed.nlm.nih.gov/dailymed/>

Normal lung sound from Thinklabs Medical, LLC, Centennial, CO at <http://www.thinklabs.com/lung-sounds>

Patient education files adapted from OSCE Skills and wikiHow at [http://www.osceskills.com/e-](http://www.osceskills.com/e-learning/subjects/explaining-the-peak-expiratory-flow-rate-technique/) [learning/subjects/explaining-the-peak-expiratory-flow-rate-technique/](http://www.osceskills.com/e-learning/subjects/explaining-the-peak-expiratory-flow-rate-technique/) and <http://www.wikihow.com/Use-a-Peak-Flow-Meter>

Pictures from Shutterstock.com

Wheeze lung sound from Wikipedia at <https://en.wikipedia.org/wiki/Wheeze>

**REFERENCES**

Global Initiative for Asthma (2016). Pocket Guide for Asthma Management and Prevention.

Downloaded from: [http://ginasthma.org/2016-gina-report-global-strategy-for-asthma-](http://ginasthma.org/2016-gina-report-global-strategy-for-asthma-management-and-prevention/) [management-and-prevention/](http://ginasthma.org/2016-gina-report-global-strategy-for-asthma-management-and-prevention/)

National Heart, Lung, Blood Institute (2007) The Expert Panel Report 3 (EPR–3) Guidelines for the Diagnosis and Management of Asthma. Downloaded from: <http://www.nhlbi.nih.gov/health-pro/guidelines/current/asthma-guidelines>)

NURSING | LEVEL: 2

Joint Commission (2016). Children’s Asthma Care. Downloaded from <https://www.jointcommission.org/childrens_asthma_care/>.

Sawicki, G. and Haver, K. (2016). Acute asthma exacerbations in children: Home/office management and severity assessment. In: UptoDate, Post TW (Ed), UptoDate, Waltham, MA. (Accessed on August 1, 2016)


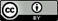


This work by the [Wisconsin Technical College System TAACCCT IV Consortium i](http://advancewisconsin.org/advance-wisconsin/it/)s licensed under a [Creative](http://creativecommons.org/licenses/by/4.0/) [Commons Attribution 4.0 International license.](http://creativecommons.org/licenses/by/4.0/)

Third party marks and brands are the property of their respective holders. Please respect the copyright and terms of use on any webpage links that may be included in this document.

This workforce product was funded by a grant awarded by the U.S. Department of Labor’s Employment and Training Administration. The product was created by the grantee and does not necessarily reflect the official position of the

U.S. Department of Labor. The U.S. Department of Labor makes no guarantees, warranties, or assurances of any kind, express or implied, with respect to such information, including any information on linked sites and including, but not limited to, accuracy of the information or its completeness, timeliness, usefulness, adequacy, continued availability, or ownership. This is an equal opportunity program. Assistive technologies are available upon request and include Voice/TTY (771 or 800-947-6644).
