# Supplementary material for: Augmented Reality Technology as a Teaching Strategy for Learning Pediatric Asthma Management: Mixed Methods Study
Source: JMIR Nurs. 2020 Dec 2;3(1):e23963. doi: 10.2196/23963 (PMC8373372; doi:10.2196/23963)
Supplement: Multimedia Appendix 3 [file nursing_v3i1e23963_app3.docx]

**Example Technology Implementation Considerations prior to using Augmented Reality Technology for Instructional activity**

| Criterion to meet | Questions to ask | Considered in current study |
| --- | --- | --- |
| Curriculum relevance | What are the instructional (teaching and learning) goals and objectives? | - Aligned with PAM content taught in graduate course |
| Technology environment | Does the environment suit for the activity (Real environment, mixed reality or virtual environment)? | - Augmented Reality (AR) layers sounds, images, and videos into real-life environments chosen |
| Course delivery format | Is the AR suitable for the class format adopted by course instructors? | - Course instructors prepared class activities using AR for flipped instruction. |
| Teaching pedagogy | Is selected AR software suitable for curricular activities or requires new development? | - AR software (ARIS) clinical scenarios were selected because it met our curricular needs |
| Pedagogy changes | Are changes needed for using scenarios upon selection to match course goals and lesson activity objectives? | - Scenario (Case study) instructions were adapted to meet the learning objectives of the activity designed for learning nursing content. - Modification to scenarios (changing QR codes) were made to suit the needs of the class instruction/student access |
| Device ownership | Are device ownership policies met per institutional guidelines? | - Although our nursing college requires students to bring their own device for their academic and clinical work activities, all students participating in the study did not own a compatible device to carry the course activities. Hence, each participating student was provided a grant funded, University-owned iPad to utilize for the duration of the study. |
| Software/Hardware requirements | Which mobile device to use? | - The ARIS application is only available for iPhone and iPad. iPads were chosen by our college. |
| Budget | What are the available funding sources? | - Department Grant funding sought for iPad purchase. - ARIS app and scenarios were free for academic use. |
| Technology authentication | How and who manages and authenticates user accounts on devices? | - Students were required during initial setup of the iPads to authenticate into the University’s mobile management system. This process associated the iPad with the individual user accounts and allowed for remote management of the devices to install updates and facilitate device recovery in case the iPad was lost or stolen. |
| Training and technology support | Does institution provide the training and support for faculty and students? | - Course faculty and students were supported by College’s IT staff. - Canvas LMS was utilized to provide handouts or content (for example, process for installing the software /technical requirements) - After initial configuration of the iPad, students were instructed to download the ARIS application from the Apple Store. Students were not limited to the ARIS application, but were granted full administrative rights to install other software as they deemed necessary on their devices. |
